# Effects of Medium-Chain Fatty Acid Glycerides on Nutrient Metabolism and Energy Utilization in Weaned Piglets

**DOI:** 10.3389/fvets.2022.938888

**Published:** 2022-06-29

**Authors:** Zhijuan Cui, Xianze Wang, Simeng Liao, Ming Qi, Andong Zha, Gang Zuo, Peng Liao, Yuguang Chen, Chun Guo, Bie Tan

**Affiliations:** ^1^College of Animal Science and Technology, Hunan Agricultural University, Changsha, China; ^2^Guangdong Provincial Key Laboratory of Animal Nutrition Control, National Engineering Research Center for Breeding Swine Industry, College of Animal Science, Institute of Subtropical Animal Nutrition and Feed, South China Agricultural University, Guangzhou, China; ^3^Laboratory of Animal Nutritional Physiology and Metabolic Process, Key Laboratory of Agro-Ecological Processes in Subtropical Region, National Engineering Laboratory for Pollution Control and Waste Utilization in Livestock and Poultry Production, Institute of Subtropical Agriculture, Chinese Academy of Sciences, Changsha, China; ^4^Center for Medical Research and Innovation, The First Hospital of Hunan University of Chinese Medicine, Changsha, China

**Keywords:** medium-chain fatty acid glycerides, enzyme activity, energy, lipid metabolism, nutrient absorption

## Abstract

Weaning stress induces the depressed digestive and absorptive capacity and insufficient intestinal energy supply. Medium-chain fatty acid glycerides have shown to improve the growth performance and intestinal barrier function of weaned piglets in the previous study. This study was aimed to investigate the regulation of medium-chain fatty acid glyceride on the nutrient absorption and energy utilization of weaned piglets. Nighty healthy weaned piglets were randomly assigned into five treatments: NP (Normal protein, normal-protein diet no antibiotics included); NC (Negative control, low-protein diet no antibiotics included); PC (Positive control, low-protein diet +75 mg/kg quinocetone, 20 mg/kg virginiamycin and 50 mg/kg aureomycin); MCT (tricaprylin + tricaprin group, low-protein diet + tricaprylin + tricaprin); GML (glycerol monolaurate group, low-protein diet + glycerol monolaurate). The results showed that GML treatment increased the ALP activity, concentrations of serine and methionine, MCT treatment increased concentrations of serine and 3-methyl-histidine but decreased TG concentration in serum. MCT and GML supplementations significantly promoted the lipase activity in the jejunum and ileum, as well as the AMP content in the ileal mucosa. GML addition significantly decreased the contents of butyric acid, isobutyric acid and total volatile fatty acid. In addition, medium chain fatty acid glycerides altered gene expressions involved in lipid metabolism, which showing the increases of AMPK2, CD36 and CGI58 and the decreases of MGAT2 and DGAT2 in the liver, as well as the increases of CD36, CGI58, MGAT2 and DGAT2 in the subcutaneous adipose tissue. These findings showed that medium-chain fatty acid glyceride can effectively improve the absorption of nutrients and lipid metabolism of piglets to meet the energy demand of weaned piglets, and then regulate the growth and development of weaned piglets.

## Introduction

Weaning stress induces villous shortening, crypt elongation, and poor digestive enzymes and nutritional transports in the gastrointestinal tract of piglets, which leading to a depressed digestive capacity and defense function of intestine (1, 2). The previous study has showed that the addition of medium-chain fatty acid glycerides to a low-protein diet could improve the growth performance and intestinal barrier function of weaned piglets (3), but its mechanism is unclear.

The depressed digestive and absorptive capacity together with the low feed intake after weaning results insufficient intestinal energy supply (4). It has been demonstrated that growth retardation is associated with decreasing capability of nutrient absorption and aberrant energy status in the intestinal mucosa of piglets in the previous study (5). The amino acids can be used as energy substrates in the small intestine (6), but typical diets based on corn and soybean meal cannot provide sufficient amino acids for protein synthesis (7). The fatty decomposed from medium-chain fatty acid glycerides can provide energy for the body quickly through β-oxidation in the liver. It has showed that medium-chain fatty acid glycerides can be rapidly oxidized *in vivo*, which providing 24–48% of the required energy for piglets (8, 9). Supplementing medium-chain fatty acid glycerides can promote the development of the intestinal tract by increasing the weight of the jejunum, promoting the renewal of epithelial cells in jejunum and accelerating the migration rate of epithelial cells along the crypt-villus axis (10). In addition, medium-chain fatty acid glycerides improved the nutrient digestibility and protein digestibility of dry matter, nitrogen and energy in piglets (11, 12).

Therefore, we hypothesis that dietary supplementation with medium-chain fatty acid glycerides can improve the utilization of nutrients, especially energy, then provide sufficient energy to intestine for the maintains of barrier function in piglets. Serum biochemistry, intestinal digestive enzyme activity and volatile fatty acids, and intestinal mucosal energy level were detected, aim to provide the scientific basis for the application of medium-chain fatty acid glycerides in a low protein diet.

## Materials and Methods

### Experimental Animal and Sample Collection

A total of 90 healthy Duroc × Landrace × Large Yorkshire piglets weaned at 21 days of age (body weight 6 ± 0.15 kg) were assigned into five treatments, with six pens per treatment and three piglets per pen. Treatments were as follows: NP (Normal protein, normal-protein diet no antibiotics included); NC (Negative control, low-protein diet no antibiotics included); PC (Positive control, low-protein diet +75 mg/kg quinocetone, 20 mg/kg virginiamycin and 50 mg/kg aureomycin); MCT (low-protein diet + tricaprylin + tricaprin); GML (low-protein diet + glycerol monolaurate). The compositions and nutritional levels of the diet were presented in the previous study (3). The medium-chain fatty acid glycerides were obtained from Deyuanshun Biological Technology Co., Ltd. (Beijing, China). The pig pens were disinfected before the start of the experiment. The thermometer and hygrometer are hung inside the pig house, and the management of the pig house is adjusted by observing the changes of temperature and humidity. Three piglets are raised in the same column, and can move and drink freely. The experiment lasted for 14 days. The growth performance of piglets was also presented in the previous study that no differences in average daily gain and feed/gain ratio among all treatments were observed (3).

On the 14th day, the serum samples were obtained and stored at −80°C as described before (13). One piglet from each pen was randomly chosen to slaughter, the samples of jejunum, ileum, liver and subcutaneous adipose tissue were collected and frozen in liquid nitrogen then stored at −80°C. The chyme from the jejunum, ileum, cecum and colon were collected in a 50 mL centrifuge tube and then transferred to the −80°C refrigerator.

### Serum Biochemical Indexes Assays

Biochemical indicators including triglyceride (TG), total cholesterol (CHOL), high-density lipoprotein (HDL) and alkaline phosphatase (ALP) were measured using an instrument (Biochemical Analytical Instrument, Beckman CX4, Beckman Coulter Inc., Brea, CA) and commercial kits (Sino-German Beijing Leadman Biotech Ltd., Beijing, China).

### Detection of Free Amino Acids in Serum

The 600 μL 8% sulfosalicylic acid was added into the serum (600 μL) and fully mixed. After being precipitated at 4°C overnight, this mixture was centrifuged at 10,000 g and 4°C for 10 min. The supernatant was detected in the ion-exchange amino acid analyzer (Hitachi L-8900 Auto-Analyzer).

### pH Determination in the Intestine

Respectively ligate the ileum, cecum, anterior colon and posterior colon and mix the contents well, the pH in the contents of four segments was measured by a Hand-held pH meter.

### Detection of Intestinal Enzyme Activity

The activities digestive enzymes including sucrase, maltase, lipase, trypsin and lactase in the jejunal and ileal chyme were measured using commercially available swine enzyme-linked immunosorbent assay (ELISA) kits according to the manufacturer's instructions (Jiangsu Meimian industrial Co., Ltd Yancheng). The enzyme activity was normalized to the protein concentration (mU/mg).

### Detection of Volatile Fatty Acids in the Colon Chyme

Accurately weigh 1 g of fresh colon chyme and mix with 5 mL deionized water for 30 min, and centrifuge the mixture at 15,000 rpm for 10 min then collect the supernatant. Repeat the above steps using 2 mL deionized water and merge the all supernatant into the 10 mL tube. The supernatant was centrifuged at 12,000 rpm for 15 min then the supernatant was transferred to 2 mL centrifuge tube according to v:v = 9:1 (900 μL supernatant + 100 μL 25% metaphosphoric acid). After mixing, place them at room temperature for 3~4 h, centrifuge, filter with 0.22 μm microporous filter membrane. Use liquid phase-gas phase analysis to detect the content of volatile fatty acids.

### Detection of ATP, ADP, AMP in the Intestinal Mucosa

Adenosine monophosphate (AMP), adenosine diphosphate (ADP) and adenosine triphosphate (ATP) in the jejunal and ileal mucosa were measured using commercially available swine enzyme-linked immunosorbent assay (ELISA) kits according to the manufacturer's instructions (Jiangsu Meimian industrial Co., Ltd Yancheng). The intestinal digestive enzyme activity was normalized to the protein concentration (mU/mg) and the AMP/ATP ratio was calculated.

### Real-Time Quantitative PCR

Expressions of monoacylglycerol acyltransferase 2 (MGAT2), diacylglycerol O-acyltransferase 2 (DGAT2), comparative gene identification-58 (CGI-58), fatty acid transferase (FAT/CD36), adenosine 5'-monophosphate (AMP)-activated protein kinase 2 (AMPK2) in the liver and subcutaneous adipose tissue were determined by real-time quantitative reverse transcriptase PCR (real-time qRT-PCR) as described previously (14). Total RNA was extracted from liver and adipose tissue with Trizol reagent (Invitgen,Thermo Fisher Science, USA) and electrophoretic on 1% agarose gel. The mass and concentration of RNA were determined by ultraviolet spectrophotometer (Nanodrop 2000 Spectrophotometer, Thermo Scientific, Courtaboeuf, France). Then AG kit (Hunan Aikerui Biological Engineering Co., Ltd.) was used to decontaminate and reverse transcribe cDNA. All PCR primers used in this study are listed in Table 1 and the primers were purchased from Shanghai Shenggong Bioengineering Co., Ltd. The relative gene expression levels were normalized to the reference gene (β-actin) were calculated by using the 2^−ΔΔCt^ method. Data were expressed as the relative values to those of piglets in NP treatment.

**Table 1:** Sequences and parameters of specific primers for real-time PCR.

**Gene**	**Sequence (5'-3')**	**Accession No**.
β-actin	Forward:CTGCGGCATCCACGAAACT	XM_021086047.1
	Reverse:AGGGCCGTGATCTCCTTCTG	
MGAT2	Forward:GCAATGGGCGACAAAGGAAG	NM_001128482.1
	Reverse:GTACCGCAGCGTCAGGTT	
DGAT2	Forward:AGTTCCCTGGCATAAAGCCCTAC	NM_001160080.1
	Reverse:AGTCTATGGTGTCCCGGTTCACA	
CGI-58	Forward:ACATGGTGCCCTACATCGAC	NM_001012407.1
	Reverse:AGTCCGAGACCTCCTCCAAA	
FAT/CD36	Forward:TGGGTTAAAACAGGCACGGA	NM_001044622.1
	Reverse:ACTGTGTGGGTCTCAGGGTC	
AMPK2	Forward:CGACGTGGAGCTGTACTGCTT	XM_021093564.1
	Revers:CATAGGTCAGGCAGAACTTGC	

*MGAT2, monoacylglycerol acyltransferase 2; DGAT2, diacylglycerol O-acyltransferase 2; CGI-58, comparative gene identification-58; FAT/CD36, Fatty acid transferase; AMPK2, Adenosine 5'-monophosphate (AMP)-activated protein kinase*.

### Statistical Analysis

Data were analyzed by an analysis of variance, using the General Linear Models procedure of the SPSS 20.0 (SPSS Inc., Chicago, IL, USA). Significant differences among treatments were evaluated using Tukey's multiple comparison tests. Results were expressed as the mean ± standard error of the mean (SEM). A value of *P* < 0.05 was considered statistically significant. Mean values and a statistical elaboration were performed by using each pen as the experimental unit (*n* = 6 per group).

## Results

### Serum Biochemical Indexes

The biochemical indexes of serum are shown in Table 2. Compared with piglets in NP treatment, the serum ALP activities in the NC and PC treatments, and TG concentration in MCT treatment were significantly decreased (*P* < 0.05). Compared with NC treatment, the serum LDH activity in GML-treated piglets was significantly decreased (*P* < 0.05). Compared with piglets in PC treatment, the serum ALP activity in GML treatment was increased, and TG concentration in MCT treatment was significantly decreased (*P* < 0.05). And the serum concentrations of CHOL and HDLI in GML-treated piglets were significantly higher than those in the other four treatments (*P* < 0.05).

**Table 2:** Effect of medium-chain fatty acid glycerides supplementation on serum biochemical indexes of piglets.

**Items**	**NP**	**NC**	**PC**	**MCT**	**GML**	***P*-value**
ALP (U/L)	260.16 ± 48.97^a^	198.16 ± 29.60^bc^	190.74 ± 24.64^c^	226.33 ± 47.56^abc^	229.83 ± 18.85^ab^	0.016
LDH (U/L)	701.83 ± 84.49^ab^	826.16 ± 149.66^a^	676.84 ± 42.75^b^	721.50 ± 110.37^ab^	614.66 ± 86.61^b^	0.023
TG (mmol/L)	0.69 ± 0.03^ab^	0.58 ± 0.22^bc^	0.85 ± 0.27^a^	0.44 ± 0.08^c^	0.79 ± 0.22^ab^	0.011
CHOL (mmol/L)	2.21 ± 0.40^b^	2.21 ± 0.23^b^	2.26 ± 0.33^b^	1.97 ± 0.22^b^	2.61 ± 0.18^a^	0.010
HDLI (mmol/L)	0.94 ± 0.17^b^	0.90 ± 0.13^b^	0.91 ± 0.09^b^	0.86 ± 0.13^b^	1.15 ± 0.19^a^	0.025

*Values are the mean ± SEM, n = 6. ^a−c^Mean values sharing different superscripts within a row differ (P < 0.05). NP, normal protein basal diet no antibiotics included; NC, low-protein basal diet no antibiotics included; PC, low-protein basal diet + antibiotics (75 mg/kg quinocetone, 20 mg/kg virginiamycin and 50 mg/kg aureomycin); MCT, low-protein basal diet + 2 kg/T tricaprylin/tricaprin; GML, low-protein basal diet + 2 kg/T glycerol monolaurate*.

### Serum Free Amino Acids

Compared with piglets in NP treatment, low-protein diets decreased some serum amino acids concentrations including serine, glycine, isoleucine, leucine and ornithine (*P* < 0.05). GML administration significantly increased serum concentrations of serine and methionine, MCT administration increased serum concentrations of serine and 3-methyl-histidine compared with the PC group (*P* < 0.05). There were no significant differences in other determined amino acid contents among these treatments (*P* > 0.05) (Table 3).

**Table 3:** Effect of medium-chain fatty acid glycerides supplementation on serum free amino acids of piglets (μg/mL).

**Item (ug/mL)**	**NP**	**NC**	**PC**	**MCT**	**GML**	***P*-value**
Serine	28.10 ± 5.16^a^	25.76 ± 6.73^a^	20.82 ± 2.34^b^	20.76 ± 2.75^b^	24.73 ± 4.24^a^	0.044
Glycine	160.58 ± 30.48^a^	133.33 ± 41.19^ab^	100.10 ± 8.70b^c^	90.08 ± 12.02^c^	121.96 ± 26.39^bc^	0.001
α-aminon-butyric acid	2.18 ± 0.70^a^	1.95 ± 0.79^a^	1.36 ± 0.28^ab^	1.11 ± 0.77^b^	2.18 ± 0.42^a^	0.024
Methionine	6.76 ± 0.93^a^	7.43 ± 1.76^a^	4.76 ± 0.77^b^	6.36 ± 1.28^a^	6.46 ± 1.28^a^	0.035
Isoleucine	20.81 ± 1.86^a^	15.73 ± 3.71^b^	15.30 ± 2.39^b^	15.91 ± 2.39^b^	17.90 ± 3.81^ab^	0.030
Leucine	36.61 ± 4.22^a^	30.96 ± 7.01^ab^	28.62 ± 3.10^b^	27.98 ± 4.52^b^	33.85 ± 5.25^ab^	0.39
Ornithine	30.13 ± 10.65^a^	18.98 ± 3.11^b^	20.78 ± 4.48^ab^	18.83 ± 5.22^b^	27.10 ± 9.70^ab^	0.044
3-methyl-histidine	2.31 ± 0.40^a^	2.41 ± 0.64^a^	1.62 ± 0.08^b^	2.31 ± 0.37^a^	1.73 ± 0.30^b^	0.007
Taurine	41.92 ± 9.60	39.63 ± 8.33	36.14 ± 9.00	40.12 ± 7.52	40.75 ± 9.78	0.863
Aspartic acid	10.40 ± 1.42	11.42 ± 2.63	9.70 ± 0.66	13.00 ± 4.53	10.17 ± 3.54	0.366
Threonine	34.08 ± 5.68	31.18 ± 9.73	22.24 ± 4.58	27.63 ± 6.87	30.35 ± 10.01	0.167
Glutamic acid	94.16 ± 17.83	90.92 ± 33.75	23.44 ± 10.48	22.68 ± 9.26	82.87 ± 20.04	0.649
Sarcosine	2.20 ± 0.77	1.42 ± 0.52	1.74 ± 0.39	1.50 ± 0.71	2.45 ± 1.04	0.094
α-aminoadipic acid	10.65 ± 0.99	9.28 ± 3.69	7.02 ± 1.22	10.63 ± 3.74	7.73 ± 2.28	0.111
Alanine	118.87 ± 31.90	113.37 ± 48.20	84.62 ± 15.37	83.02 ± 11.59	109.37 ± 24.02	0.156
Citrulline	9.32 ± 1.15	8.15 ± 1.68	6.56 ± 0.69	6.88 ± 2.48	8.43 ± 1.77	0.065
Valine	30.87 ± 4.46	25.36 ± 8.38	25.42 ± 3.21	24.53 ± 5.71	28.53 ± 4.77	0.292
Cysteine	9.97 ± 2.72	7.23 ± 1.88	8.20 ± 1.61	7.88 ± 1.72	10.95 ± 4.20	0.120
Cystic acid	2.71 ± 0.63	2.56 ± 0.79	2.02 ± 0.46	2.30 ± 0.55	2.32 ± 0.44	0.365
Tyrosine	33.68 ± 4.85	27.87 ± 7.17	29.22 ± 4.82	28.30 ± 11.17	28.22 ± 7.46	0.658
Phenylalanine	21.53 ± 2.65	20.58 ± 5.27	18.96 ± 3.42	19.83 ± 2.25	20.68 ± 1.68	0.753
β-alanine	4.63 ± 0.92	3.80 ± 1.40	3.98 ± 1.19	3.31 ± 0.86	3.45 ± 0.64	0.229
Ethanolamine	1.43 ± 0.10	1.30 ± 0.17	1.14 ± 0.23	1.02 ± 0.15	1.47 ± 0.56	0.068
Lysine	71.88 ± 10.32	70.67 ± 23.84	60.58 ± 11.61	64.02 ± 18.29	65.05 ± 18.94	0.801
Carnosine	3.75 ± 1.48	1.95 ± 1.65	5.22 ± 2.78	4.53 ± 2.77	4.60 ± 1.41	0.784
Arginine	59.75 ± 7.27	51.08 ± 11.97	41.82 ± 2.55	47.60 ± 14.60	52.78 ± 13.85	0.140
Proline	73.08 ± 11.67	67.13 ± 24.81	55.10 ± 10.35	55.20 ± 7.55	63.47 ± 12.89	0.213

*Values are the mean ± SEM, n = 6. ^a−c^Mean values sharing different superscripts within a row differ (P < 0.05). NP, normal protein basal diet no antibiotics included; NC, low-protein basal diet no antibiotics included; PC, low-protein basal diet + antibiotics (75 mg/kg quinocetone, 20 mg/kg virginiamycin and 50 mg/kg aureomycin); MCT, low-protein basal diet + 2 kg/T tricaprylin/tricaprin; GML, low-protein basal diet + 2 kg/T glycerol monolaurate*.

### The pH of Intestinal Chyme

**Figure 1:**
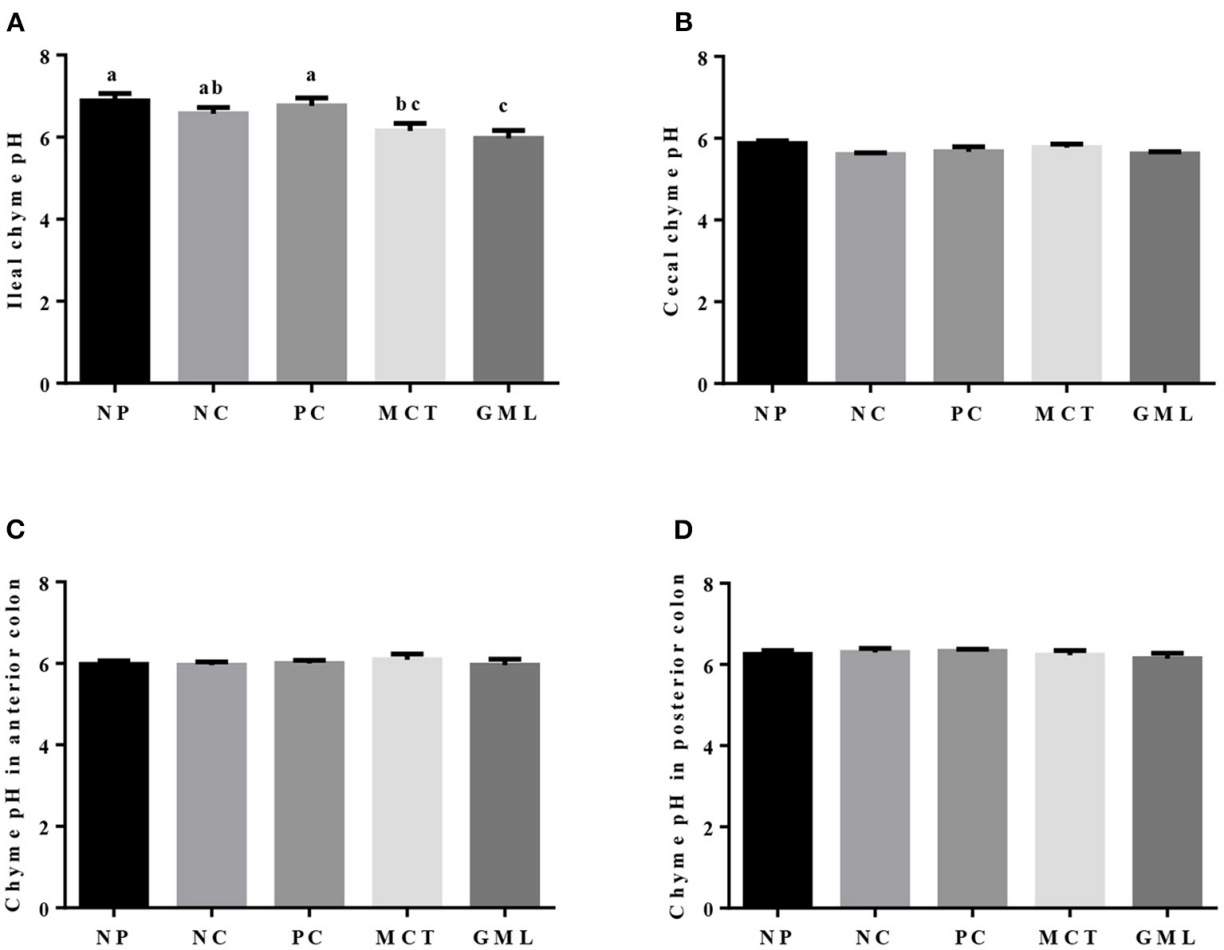
The pH in the chyme of ileum **(A)**, cecum **(B)**, anterior colon **(C)** and posterior colon **(D)**. ^a−*c*^Mean values sharing different superscripts within a row differ (*P* < 0.05). Values are the mean ± SEM, *n* = 6. NP, normal protein basal diet no antibiotics included; NC, low-protein basal diet no antibiotics included; PC, low-protein basal diet + antibiotics (75 mg/kg quinocetone, 20 mg/kg virginiamycin and 50 mg/kg aureomycin); MCT, low-protein basal diet + 2 kg/T tricaprylin/tricaprin; GML, low-protein basal diet + 2 kg/T glycerol monolaurate.

The pH value of intestinal chyme is shown in Figure 1. MCT and GML supplementations had no significant effect on the pH in the chyme of cecum, anterior colon and posterior colon of piglets (*P* > 0.05). Compared with piglets of NP and PC treatments, the pH of ileal chyme was significantly decreased in response to MCT and GML administrations (*P* < 0.05).

### Intestinal Digestive Enzyme Activity

**Figure 2:**
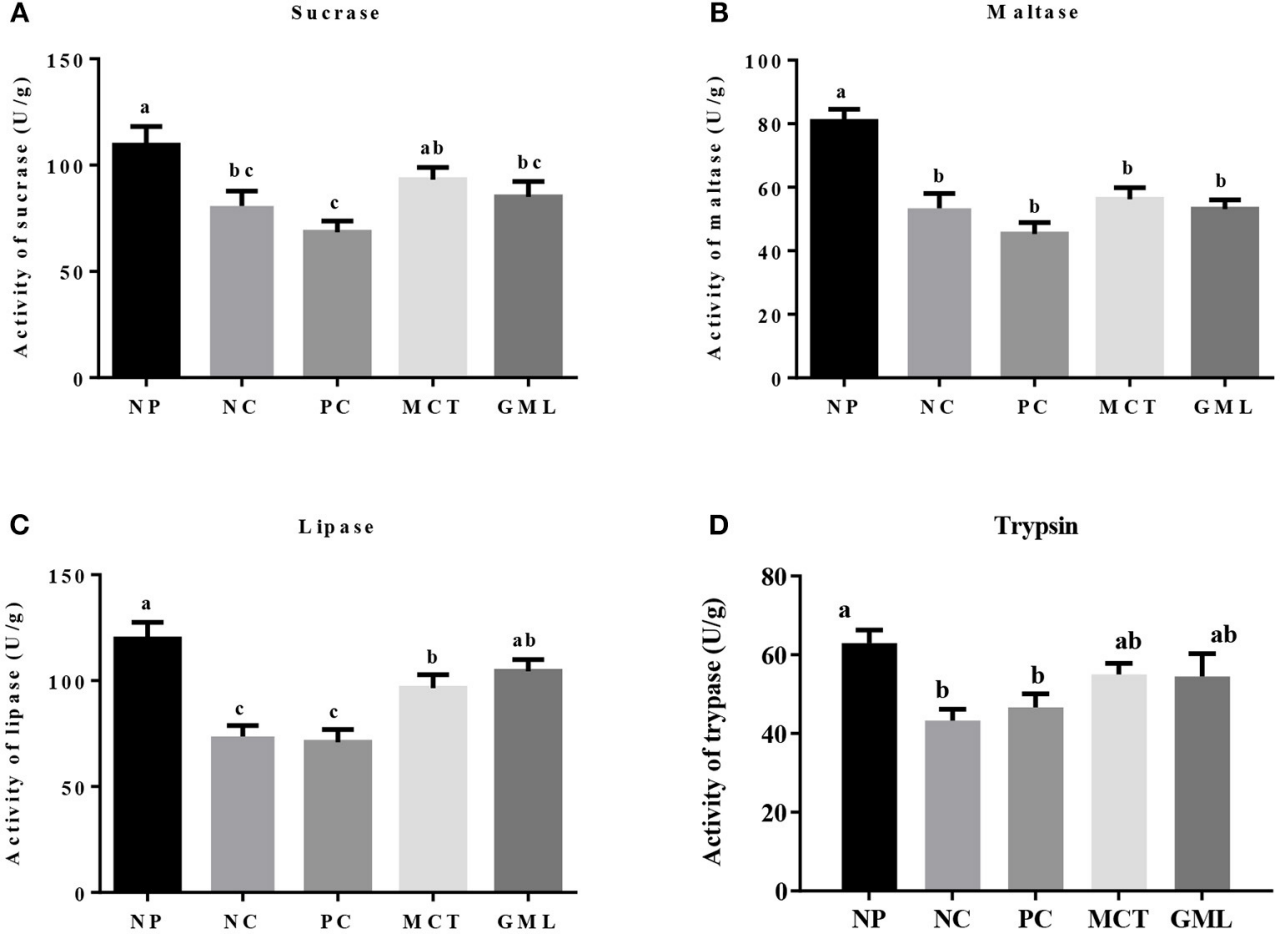
**(A–D)** The jejunal digestive enzyme activity. ^a−*c*^Mean values sharing different superscripts within a row differ (*P* < 0.05). Values are the mean ± SEM, *n* = 6. NP, normal protein basal diet no antibiotics included; NC, low-protein basal diet no antibiotics included; PC, low-protein basal diet + antibiotics (75 mg/kg quinocetone, 20 mg/kg virginiamycin and 50 mg/kg aureomycin); MCT, low-protein basal diet + 2 kg/T tricaprylin/tricaprin; GML, low-protein basal diet + 2 kg/T glycerol monolaurate.

In the jejunum, compared with piglets of NP treatment, the activities of sucrase, maltase, lipase and trypsin in NC and PC treatments were significantly decreased (*P* < 0.05). But there were no differences in the sucrose and trypsin activities between NP and MCT treatments, and no differences in the lipase and trypsin activities between NP and GML treatments (*P* > 0.05). MCT and GML supplementations significantly promoted the lipase activity compared with NC and PC treatments (*P* < 0.05) (Figure 2).

**Figure 3:**
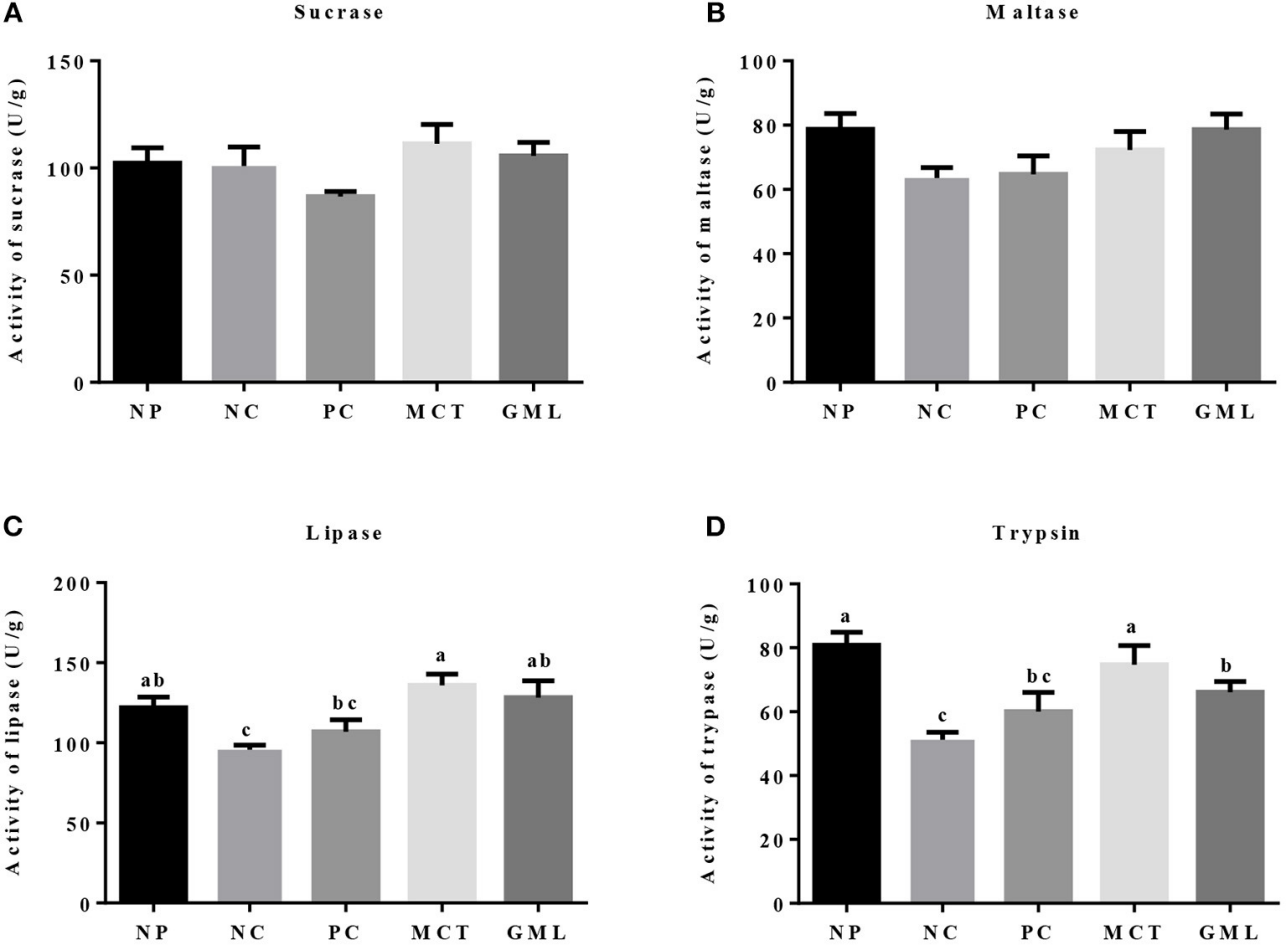
**(A–D)** The ileal digestive enzyme activity. ^a−*c*^Mean values sharing different superscripts within a row differ (*P* < 0.05). Values are the mean ± SEM, *n* = 6. NP, normal protein basal diet no antibiotics included; NC, low-protein basal diet no antibiotics included; PC, low-protein basal diet + antibiotics (75 mg/kg quinocetone, 20 mg/kg virginiamycin and 50 mg/kg aureomycin); MCT, low-protein basal diet + 2 kg/T tricaprylin/tricaprin; GML, low-protein basal diet + 2 kg/T glycerol monolaurate.

In the ileum, the lipase and trypsin activities in piglets of MCT treatment were significantly higher than that of NC and PC treatments (P < 0.05). GML addition increased the activities of lipase and trypsin compared with NC treatment (*P* < 0.05). There were no differences in sucrase and maltase activities among five treatments, as well as lipase and trypsin activities among NP, MCT and GML treatments (*P* > 0.05) (Figure 3).

### Volatile Fatty Acids in Colonic Chyme

Compared with piglets of NP treatment, MCT and GML treatments had no significant effects on all determined volatile fatty acids in colonic chime (*P* > 0.05) except for the isobutyric acid content in GML treatment (*P* < 0.05). Compared with NC group, GML addition significantly decreased the contents of butyric acid, isobutyric acid and total volatile fatty acid (*P* < 0.05) (Table 4).

**Table 4:** Effect of medium-chain fatty acid glycerides supplementation on volatile fatty acids contents in the colon chyme of piglets (μg/g).

**Items**	**NP**	**NC**	**PC**	**MCT**	**GML**	***P*-value**
Acetic acid	4638.46 ± 1252.43	5537.77 ± 234.63	4877.52 ± 458.97	5343.67 ± 478.28	3901.40 ± 1054.44	0.080
Propionic acid	1955.25 ± 230.15	2544.35 ± 551.27	2646.55 ± 708.70	2312.31 ± 254.77	2059.21 ± 125.11	0.160
Isobutyric acid	73.21 ± 33.4^a^	65.71 ± 9.01^a^	91.88 ± 27.4^a^	91.80 ± 13.8^a^	32.90 ± 3.29^b^	0.006
Butyric acid	734.79 ± 215.91^b^	1691.68 ± 449.59^a^	1188.74 ± 355.18^ab^	1243.03 ± 459.84^ab^	906.79 ± 500.98^b^	0.042
Isovaleric acid	116.61 ± 65.96a^bc^	95.23 ± 6.89^bc^	168.12 ± 67.96^a^	158.77 ± 28.04^ab^	47.58 ± 6.16^c^	0.010
Valeric acid	171.08 ± 64.95	640.01 ± 312.80	517.11 ± 348.65	455.82 ± 276.23	285.21 ± 212.28	0.141
Total volatile fatty acids	8527.98 ± 383.19^bc^	10574.75 ± 1138.31^a^	9489.93 ± 1005.34^ab^	9605.42 ± 732.40^ab^	7920.63 ± 315.03^c^	0.020

*Values are the mean ± SEM, n = 6. ^a−c^Mean values sharing different superscripts within a row differ (p < 0.05). NP, normal protein basal diet no antibiotics included; NC, low-protein basal diet no antibiotics included; PC, low-protein basal diet + antibiotics (75 mg/kg quinocetone, 20 mg/kg virginiamycin and 50 mg/kg aureomycin); MCT, low-protein basal diet + 2 kg/T tricaprylin/tricaprin; GML, low-protein basal diet + 2 kg/T glycerol monolaurate*.

### Energy Level of the Intestinal Mucosa

**Figure 4:**
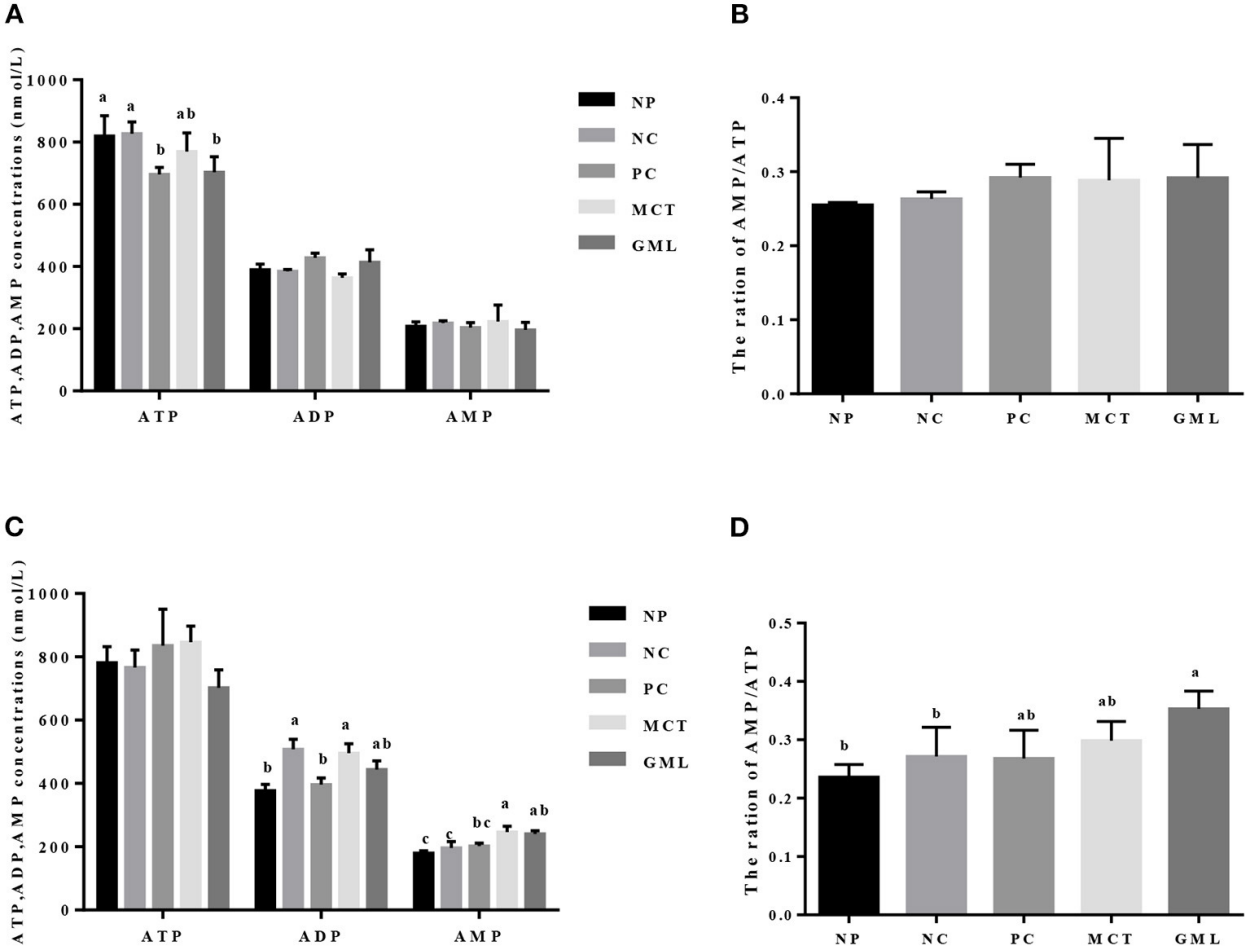
The energy level in the jejunal mucosa **(A,B)** and ileal **(C,D)** mucosa. ^a−*c*^Mean values sharing different superscripts within a row differ (*P* < 0.05). Values are the mean ± SEM, *n* = 6 per group. NP, normal protein basal diet no antibiotics included; NC, low-protein basal diet no antibiotics included; PC, low-protein basal diet + antibiotics (75 mg/kg quinocetone, 20 mg/kg virginiamycin and 50 mg/kg aureomycin); MCT, low-protein basal diet + 2 kg/T tricaprylin/tricaprin; GML, low-protein basal diet + 2 kg/T glycerol monolaurate.

The energy level of the intestinal mucosa is shown in Figure 4. Compared with piglets of NP and NC treatments, GML addition decreased the ATP level in the jejunal mucosa (*P* < 0.05).

In the ileal mucosa, the ADP concentration in MCT treatment was significantly higher than that in NP and PC treatments (*P* < 0.05), but there were no significant differences among GML, NP, NC and PC treatments (*P* > 0.05). MCT administration significantly increased the AMP content compared with NP, NC and PC treatments, while GML treatment increased the AMP content and the ratio of AMP/ATP compared with NP and NC treatments (*P* < 0.05).

### The MRNA Expressions of Genes Associated With Lipid Sensing in the Liver and Adipose Tissue

**Figure 5:**
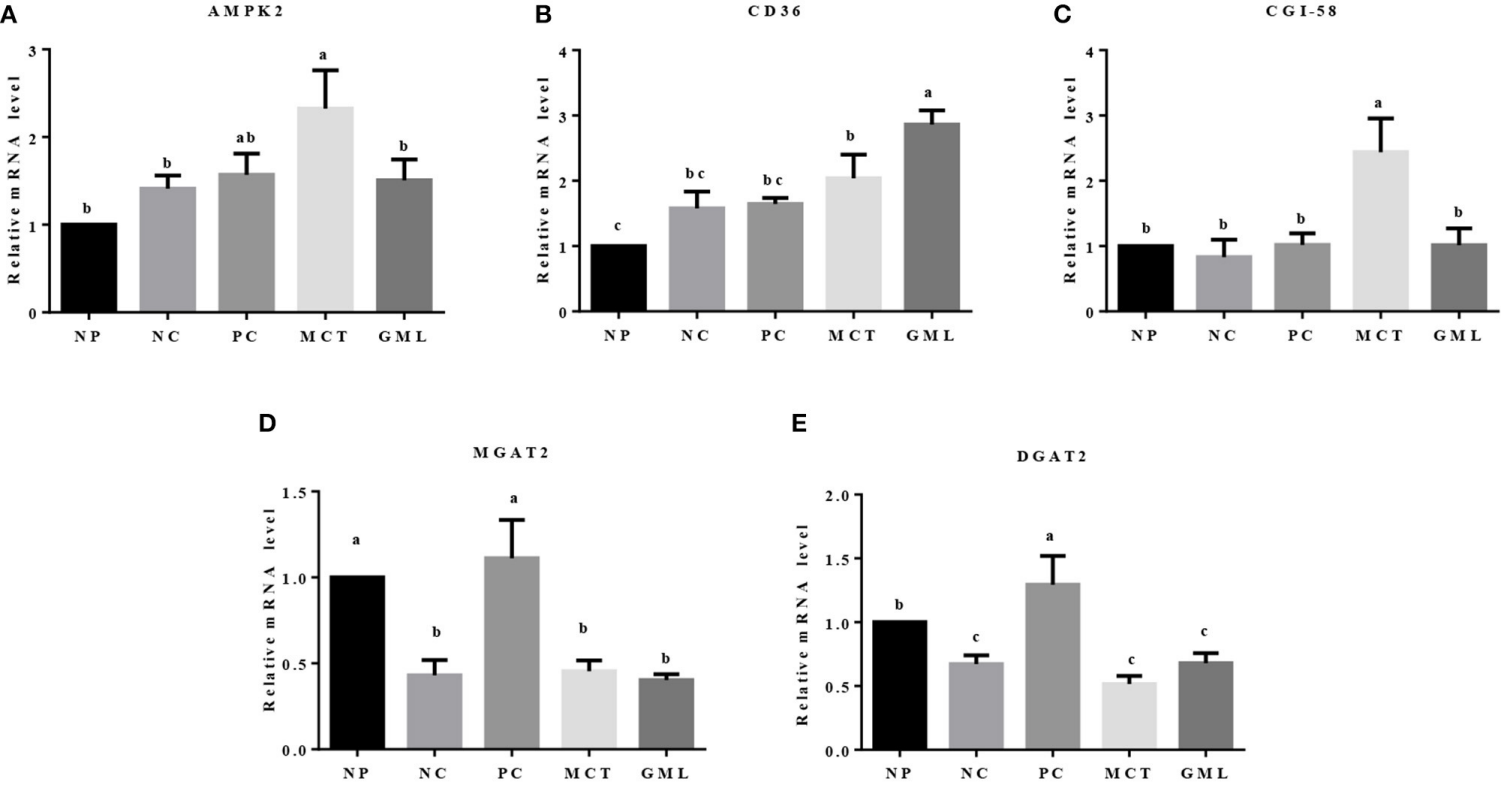
**(A–E)** The mRNA expressions of genes associated with lipid sensing in the liver. ^a−*c*^Mean values sharing different superscripts within a row differ (*P* < 0.05). Values are the mean ± SEM, *n* = 6. NP, normal protein basal diet no antibiotics included; NC, low-protein basal diet no antibiotics included; PC, low-protein basal diet + antibiotics (75 mg/kg quinocetone, 20 mg/kg virginiamycin and 50 mg/kg aureomycin); MCT, low-protein basal diet + 2 kg/T tricaprylin/tricaprin; GML, low-protein basal diet + 2 kg/T glycerol monolaurate.

In the liver, compared with the piglets of NP treatment, MCT treatment increased the mRNA expressions of AMPK-2, CD36, CGI-58 but decreased the MGAT2 and DGAT2 expressions (*P* < 0.05). MCT addition also increased the expressions of AMPK-2 and CGI-58 mRNA compared with NC treatment, and decreased the MGAT2 and DGAT2 expressions compared with PC treatment (*P* < 0.05). In piglets of GML treatment, the expression of CD36 mRNA was significantly higher than that in NP, NC and PC treatments, the MGAT2 and DGAT2 expressions were lower than that in NP and PC treatments (*P* < 0.05) (Figure 5).

**Figure 6:**
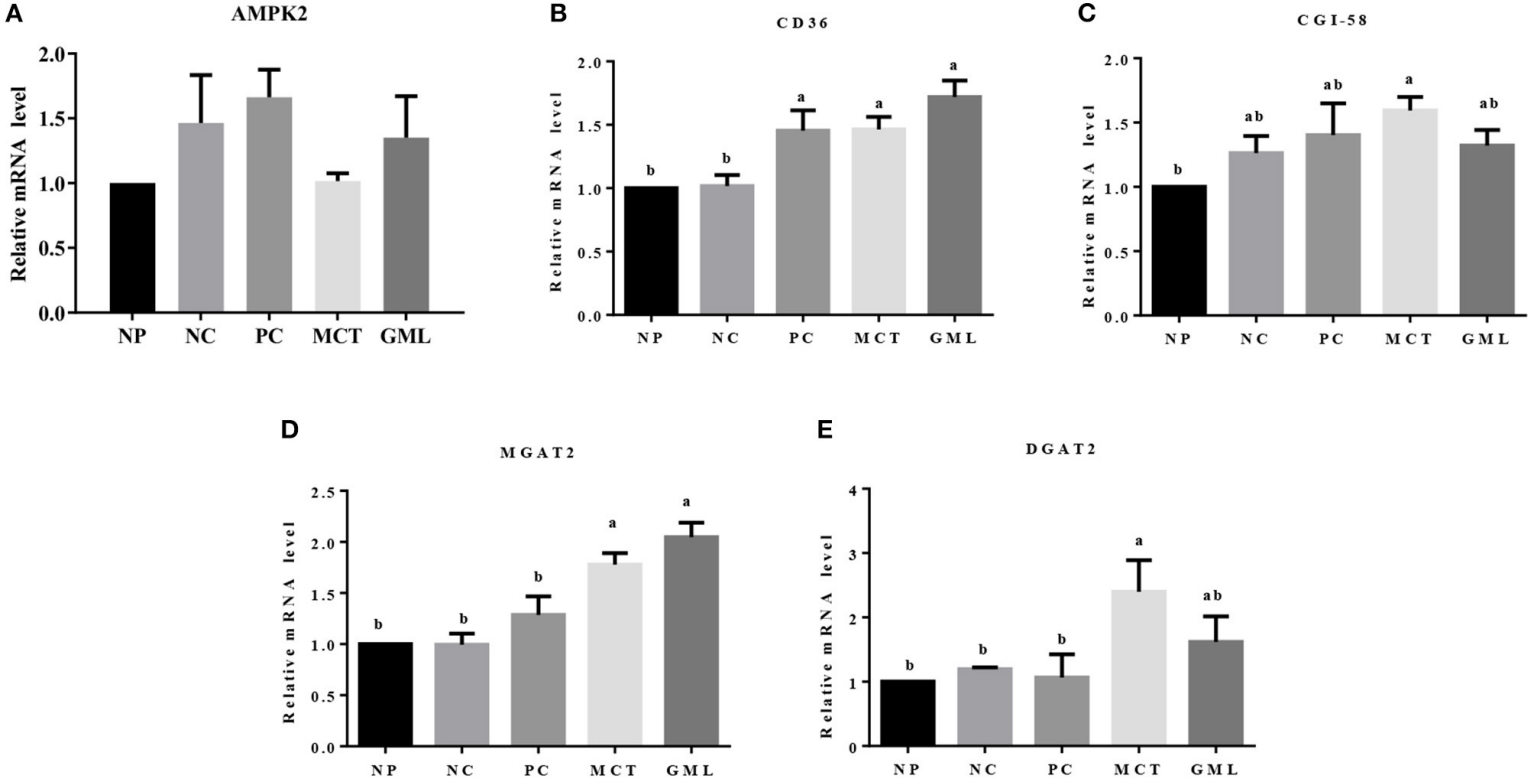
**(A–E)** The mRNA expressions of genes associated with lipid sensing in the subcutaneous adipose tissue. ^a−*c*^Mean values sharing different superscripts within a row differ (*P* < 0.05). Values are the mean ± SEM, *n* = 6. NP, normal protein basal diet no antibiotics included; NC, low-protein basal diet no antibiotics included; PC, low-protein basal diet + antibiotics (75 mg/kg quinocetone, 20 mg/kg virginiamycin and 50 mg/kg aureomycin); MCT, low-protein basal diet + 2 kg/T tricaprylin/tricaprin; GML, low-protein basal diet + 2 kg/T glycerol monolaurate.

In the subcutaneous adipose tissue, MCT and GML administrations significantly increased the expressions of CD36 and MGAT2 mRNA compared with NP and NC treatments (*P* < 0.05). Compared with PC treatment, MCT and GML supplementations had no effects on the expressions AMPK2, CD36 and CGI-58 (*P* > 0.05), but showed an increased MGAT2 expression (*P* < 0.05) (Figure 6).

## Discussion

In the previous studies, it has shown that the medium-chain fatty acid glyceride can improve the growth performance and intestinal function of piglets. In this study, we further found that medium-chain fatty acid glyceride can not only affect the blood index, intestinal enzyme activity and energy state of piglets, but also change the fat metabolism of liver and adipose tissue. These changes may make piglets better to adapt to weaning stress, and thus improve their growth.

Firstly, the medium-chain fatty acid glyceride has changed the biochemical index and amino acid in serum. It is reported that the serum biochemistry can reflect the functional changes of some tissues and organs and the ability to digest and absorb nutrients (15). The ALP widely exists in the liver, intestine, serum as well as other tissues, and is considered to be a membrane transporter (16). Thus, the liver damage can be judged by measuring ALP activity in serum. The MCT and GML have increased the activity of serum ALP, but there was no significant difference. LDH is an important enzyme involved in glycolysis and gluconeogenesis. When piglets are exposed to weaning stress, the LDH activity in the blood increases significantly and damages the intestinal barrier (17, 18). The serum LDH activity decreased significantly in GML-treatment, suggesting that GML can alleviate weaning stress and ensure the health of weaned piglets. The serum TG, CHOL, HDL-C and LDL-C are important indexes to measure lipid metabolism. It is well-known that lower HDL-C and higher TG and CHOL levels are positive correlation with cardiovascular disease (19). The medium-chain fatty acid can reduce fat deposition in mice and increase TG content in serum (20). However, medium-chain fatty acid glycerides reversely reduced TG content in this study, which can effectively prevent cardiovascular disease (21). CHOL and HDL-C are very important for lipid transport. As a protective factor against atherosclerosis, HDL-C is recognized as “good cholesterol.” In this study, GML increased the content of CHOL and HDL-C in serum, but LDL-C had no obvious effect, suggesting that GML regulated lipid metabolism and promoted lipid transport mainly by increasing the level of HDL-C. Free amino acids in serum participate in the metabolism of amino acids and the synthesis of protein, which can reflect the nutritional status of the body (22, 23). The medium-chain fatty acid can be rapidly oxidized and supply energy in liver mitochondria, and can increase the intake of calcium and amino acids to promote the synthesis of intracellular protein (23). Our study showed that MCT significantly increased the levels of serum serine and 3-methyl-histidine, which may be related to the increase of the content of medium-chain fatty acids in serum. In addition, the serine and methionine in serum of piglets were significantly increased by adding medium-chain fatty acid glycerides, which indicated that the addition of medium-chain fatty acid glycerides had improved the utilization rate of amino acids and helped piglets to synthesize protein.

Secondly, the medium-chain fatty acid glyceride has lowered intestinal acidity, enhanced digestive enzymes as well as improved energy status. The previous results highlighted reduced expression of genes encoding enzyme, transporter, and receptor, and their related pathway involved in nutrients metabolism in the jejunal mucosa of growth retardation pigs, indicating that the digestion and absorption of nutrients is closely related to the weight gain of piglets (24). Acidity in the gastrointestinal tract of piglets can maintain electrolyte balance and provide a suitable acidic environment for digestive enzymes. At the same time, the intestinal pH value is an important factor to determine the intestinal microecological conditions while *lactic acid bacteria* are not affected at lower pH (25). The previous studies showed that medium-chain fatty acids can lower the pH of intestinal tract, which may change the microbial system of the piglet intestinal tract (26, 27). Consistent with the results of previous studies, the MCT and GML significantly decreased the pH of piglet ileum when compared with PC-treatment. Our study showed that medium-chain fatty acid glyceride could keep the small intestine of piglets at low acidity, maintain the good digestion ability of piglets and promote the absorption of nutrients. Meanwhile, weaning can inhibit the activity of intestinal digestive enzymes and reduce the function of digestion and absorption of nutrients (28, 29). However, the addition of MCT and GML significantly increased the activities of lipase and trypsin in jejunum and ileum, and the medium-chain fatty acid glycerides were hydrolyzed to medium-chain fatty acids, which could be further oxidized in the liver to provide energy. Volatile fatty acids are mainly fermented by anaerobic microorganisms using soluble carbohydrates (30). GML can significantly reduce the contents of isobutyric acid, butyric acid and total acid in colon surimi, which may promote the absorption of nutrients in the small intestine and reduce the fermentation of nutrients in the colon. This is consistent with the research of Hanczakowska et al., the total acid concentration in cecum decreased with the addition of medium-chain fatty acids, suggesting that the utilization of nutrients in piglets was improved (31). Early weaning reduces the production of intestinal epithelial energy of piglets, which leads to the lack of energy in early weaning period, thus inhibiting the growth and development of weaned piglets (4). In previous studies, adding 10% fat to the diet had no significant effect on the growth performance of piglets (32, 33). However, Odle et al. found that medium-chain fatty acid glycerides can provide about 20%~40% of the energy demand for the piglets (8). ATP is an unstable high-energy compound, which can be converted into ADP during hydrolysis and release a lot of energy. Previous studies have shown that AMPK plays an important role in regulating intestinal energy utilization of piglets and can restore energy homeostasis in intestines and liver of piglets by activating AMPK pathway (5, 13). In this study, GML decreased the concentration of ATP in jejunum while MCT increased the concentration of ADP in ileum. The ATP produced by the oxidation of medium chain fatty acids is rapidly utilized by the intestinal tract, so the hydrolysis of ATP in the intestinal tract increases, resulting in the increase of ADP. With the increase of ATP utilization, the ratio of AMP/ATP is increased, so it is possible to regulate intestinal energy balance by stimulating AMPK.

Lastly, medium-chain fatty acid glycerides can reduce the synthesis of triglycerides in the liver and reversely promote lipid deposition in adipose tissue. The fat deposition in non-adipose tissue is considered to be ectopic fat deposition, which can cause metabolic disorders and organ dysfunction (34). MCT could increase CGI-58 and AMPK expression level to regulate the lipid metabolism in the liver by promoting the hydrolysis of triglycerides. The AMPK, ATGL and CGI-58 play a key role in the regulation of lipid metabolism (13, 35), which could promote the catabolism of fat stored in fat and non-fat tissues. Moreover, the ATP produced by β oxidation of fatty acids in liver is rapidly utilized to activate AMPK. In our study, for reducing the utilization of ATP, AMPK promoted the hydrolysis of triglyceride in liver and inhibited the synthesis of triglyceride. It has been reported that the medium-chain fatty acid glyceride is difficult to store because they will be oxidized rapidly *in vivo*, so they can reduce the deposition of adipose tissue (36). The previous studies found that medium-chain fatty acid glyceride can reduce the weight gain and body fat deposition of mice more than long-chain fatty acid glyceride (37, 38). When sufficient energy is obtained in the body, excess carbohydrate and fat will be converted into triglyceride and stored in adipose tissue, and CD36, MGAT2 and DGAT2 play an important role in this pathway (39). In this study, GML can significantly increase the expression level of CD36 while MCT can significantly increase the expression level of CD36, MGAT2, DGAT2 in adipose tissue. The results showed that medium-chain fatty acid glyceride promoted the intake of fatty acids and increased the synthesis of triglycerides in adipose tissue. It is possible that medium-chain fatty acid glycerides can provide sufficient energy supply for piglets, and nutrients obtained from the diets of piglets are finally stored in fatty tissues in the form of triglycerides. It is also possible that a small part of absorbed medium-chain fatty acid is stored in adipose tissue, or it is related to the growth of long-chain fatty acids and the further synthesis of triglycerides for chain extension.

## Conclusions

In summary, this study provided new evidence for the application of medium-chain fatty acid triglycerides in low-protein diets for piglets. Specifically, dietary supplementations with medium-chain fatty acid triglycerides can increase the ALP activity and amino acids concentrations in serum, promote the lipase activity and energy levels in the small intestine but decrease the volatile fatty acid production in the colon. In addition, medium-chain fatty acid glycerides altered gene expressions involved in lipid metabolism in the liver and subcutaneous adipose tissue. These findings showed that medium-chain fatty acid glyceride can effectively improve the absorption of nutrients and lipid metabolism of piglets to meet the energy demand of weaned piglets, and then regulate the growth and development of weaned piglets.

## Data Availability Statement

The raw data supporting the conclusions of this article will be made available by the authors, without undue reservation.

## Ethics Statement

The animal study was reviewed and approved by the Institute of Subtropical Agriculture, Chinese Academy of Sciences.

## Author Contributions

ZC and BT designed the experiments. ZC, XW, and CG conducted the experiments. SL, MQ, AZ, and GZ helped with animal experiments. ZC analyzed the data and wrote the original draft. PL, YC, and BT revised the manuscript. All authors read and approved the final manuscript.

## Funding

This research was supported by the National Natural Science Foundation of China (U20A2054, 32072745, 32102571, and 32130099), National Key R&D Program (2021YFD1301004, 2021YFD1301005, and 2021YFD1300401), China Agriculture Research System of MOF and MARA.

## Conflict of Interest

The authors declare that the research was conducted in the absence of any commercial or financial relationships that could be construed as a potential conflict of interest.

## Publisher's Note

All claims expressed in this article are solely those of the authors and do not necessarily represent those of their affiliated organizations, or those of the publisher, the editors and the reviewers. Any product that may be evaluated in this article, or claim that may be made by its manufacturer, is not guaranteed or endorsed by the publisher.

## Abbreviations

ADP: adenosine diphosphate
ALP: alkaline phosphatase
AMP: adenosine monophosphate
AMPK2: Adenosine 5'-monophosphate (AMP)-activated protein kinase
ATP: adenosine triphosphate
CD36: Fatty acid transferase
CGI-58: Comparative gene identification-58
CHOL: total cholesterol
CPT2: carnitine palmitoyl transferase 2
CRE: corticotropin-releasing factor
DGAT2: Diacylglycerol acyltransferase 2
FABP1: fatty acid-binding protein 1
GPR: G protein-coupled receptor
H3K9: histone-3 lysine-9
HDL: high-density lipoprotein
LDH: lactate dehydrogenase
LPL: lipoprotein lipase
MGAT2: Monoacylglycerol Acyltransferase 2
TG: triglyceride.
